# Soft Perches in an Aviary System Reduce Incidence of Keel Bone Damage in Laying Hens

**DOI:** 10.1371/journal.pone.0122568

**Published:** 2015-03-26

**Authors:** Ariane Stratmann, Ernst K. F. Fröhlich, Alexandra Harlander-Matauschek, Lars Schrader, Michael J. Toscano, Hanno Würbel, Sabine G. Gebhardt-Henrich

**Affiliations:** 1 Research Center for Proper Housing: Poultry and Rabbits (ZTHZ), Division of Animal Welfare, University of Bern, Bern, Switzerland; 2 Research Center for Proper Housing: Poultry and Rabbits (ZTHZ), Federal Food Safety and Veterinary Office, Bern, Switzerland; 3 Animal and Poultry Science, University of Guelph, Guelph, Ontario, Canada N1G2W1; 4 Institute of Animal Welfare and Animal Husbandry, Friedrich-Loeffler-Institut, Celle, Germany; Institut Pluridisciplinaire Hubert Curien, FRANCE

## Abstract

Keel bone fractures and deviations are one of the major welfare and health issues in commercial laying hens. In non-cage housing systems like aviaries, falls and collisions with perches and other parts of the housing system are assumed to be one of the main causes for the high incidence of keel bone damage. The objectives of this study were to investigate the effectiveness of a soft perch material to reduce keel bone fractures and deviations in white (Dekalb White) and brown laying hens (ISA Brown) kept in an aviary system under commercial conditions. In half of 20 pens, all hard, metal perches were covered with a soft polyurethane material. Palpation of 20 hens per pen was conducted at 18, 21, 23, 30, 38, 44 and 64 weeks of age. Production data including egg laying rate, floor eggs, mortality and feed consumption were collected over the whole laying period. Feather condition and body mass was assessed twice per laying period. The results revealed that pens with soft perches had a reduced number of keel bone fractures and deviations. Also, an interaction between hybrid and age indicated that the ISA hybrid had more fractured keel bones and fewer non-damaged keel bones compared with the DW hybrid at 18 weeks of age, a response that was reversed at the end of the experiment. This is the first study providing evidence for the effectiveness of a soft perch material within a commercial setting. Due to its compressible material soft perches are likely to absorb kinetic energy occurring during collisions and increase the spread of pressure on the keel bone during perching, providing a mechanism to reduce keel bone fractures and deviations, respectively. In combination with genetic selection for more resilient bones and new housing design, perch material is a promising tool to reduce keel bone damage in commercial systems.

## Introduction

Keel bone damage—specifically bone fractures and bone deviations—is a major welfare problem in commercial laying hens and possibly the greatest welfare issue egg production is currently facing [1,2]. Different studies report frequencies of keel bone damage varying between 56% and 97% of affected birds per flock [3, 4, 5] indicating that millions of birds are affected. Causes for keel bone damage are assumed to originate from two factors: 1) genetic selection for increased egg production and 2) inappropriate housing design [6]. Genetic selection for increased egg laying rate is believed to result in loss of structural bone mass, which leads to weakened bones prone to fracture [7]. The role of genetic selection in causing keel bone damage is also supported by a higher incidence of fractures in commercial lines compared with traditional breeds [8]. In terms of housing design, the shift away from battery cages (Switzerland in 1992, EU in 2012) to alternative housing systems such as aviaries allows for greater freedom to move and fly, which likely leads to a higher incidence of keel bone fractures due to high energy collisions in these systems [9]. Specifically, housing systems containing perches have a greater prevalence of keel bone fractures and perch height is positively correlated with the prevalence of fractures [5] suggesting the influential role of collisions. The two categories of keel bone damage—fractures and deviations—likely have different prevalence and origins: fractures are assumed to be caused by short-term, high energetic impacts, which occur during collisions with perches or other housing structures [10]. In contrast, keel bone deviations are assumed to result from extended perching behaviour and related to long-term pressure on the keel bone [11, 12]. There is evidence that keel bone fractures are associated with pain, indicated by behavioural differences between birds with and without old keel bone fractures in mobility tests (e.g. descending from different perch heights) [13, 14, 15]. Moreover, Gentle [16] found that birds possess nociceptive C- fibers that are similar to those of humans and thought to be a prerequisite for the perception of pain, suggesting that birds are capable of experiencing pain similar to humans. Keel bone fractures are also associated with decreased egg production [13] and have been shown to be related with increased mortality as well [17] and thus probably have a detrimental effect on economic return [18]. Although perches are a likely source of keel bone damage, they are an important resource for laying hens and essential for roosting, a highly valued activity [19, 20], originating from the ancestral red jungle fowl [21]. In non-cage housing systems such as multi-tiered aviaries, perches are important because birds use them to rest, move to different tiers in the system, to access feeders and drinkers as well as for night-time roosting. Typically, only one perch type per hen house is available in commercial systems, making it important to consider perch characteristics that are beneficial for the birds’ health and welfare. Assuming that perches likely relate to the high incidence of keel bone damage, studies on pressure load on keel bones while hens are resting on different types of perches showed that using alternative perch material such as cushioned perches can minimize pressure load on the keel bone and increase its contact area when a hen is perching [12]. However, whether soft perches also reduce keel bone damage within commercial systems has not been investigated yet.

Therefore, the main objective of this study was to evaluate the effectiveness of a soft, polyurethane cover for perches to reduce the incidence of keel bone fractures and deviations in two layer strains kept in a commercial aviary system. Our reasoning was that soft perches would provide a buffering effect and thus reduce the force that occurs at the keel bone during high energy collisions, reducing the number of keel bone fractures. Additionally, soft perches were expected to reduce local pressure load on the keel bone during perching resulting in reduced incidence of keel bone deviation. We thus hypothesized that fewer keel bone fractures and deviations would occur in pens where perches were covered with a soft material compared with hard, metal perches.

## Methods

### Ethics statement

All procedures were approved by the Cantonal Veterinary Office of Berne, Switzerland (Cantonal license number BE 99/11) and all corresponding ethical guidelines were followed (S1 File). Before the study began, criteria were established as to when experimental animals would be euthanized when welfare was seen to be compromised. Criteria included inability to walk/ perform natural motion and/or gross or open lesions. No focal animals met these criteria, though several non-study animals within the overall flock were euthanized by the animal care staff for these reasons. Euthanasia was performed by a concussive blow to the animal’s head followed by cervical dislocation, a procedure accepted as a legal form of killing for laying hens in Switzerland (animal protection guideline 800.116–3.01, BLV).

### Birds and management

**Fig 1:**
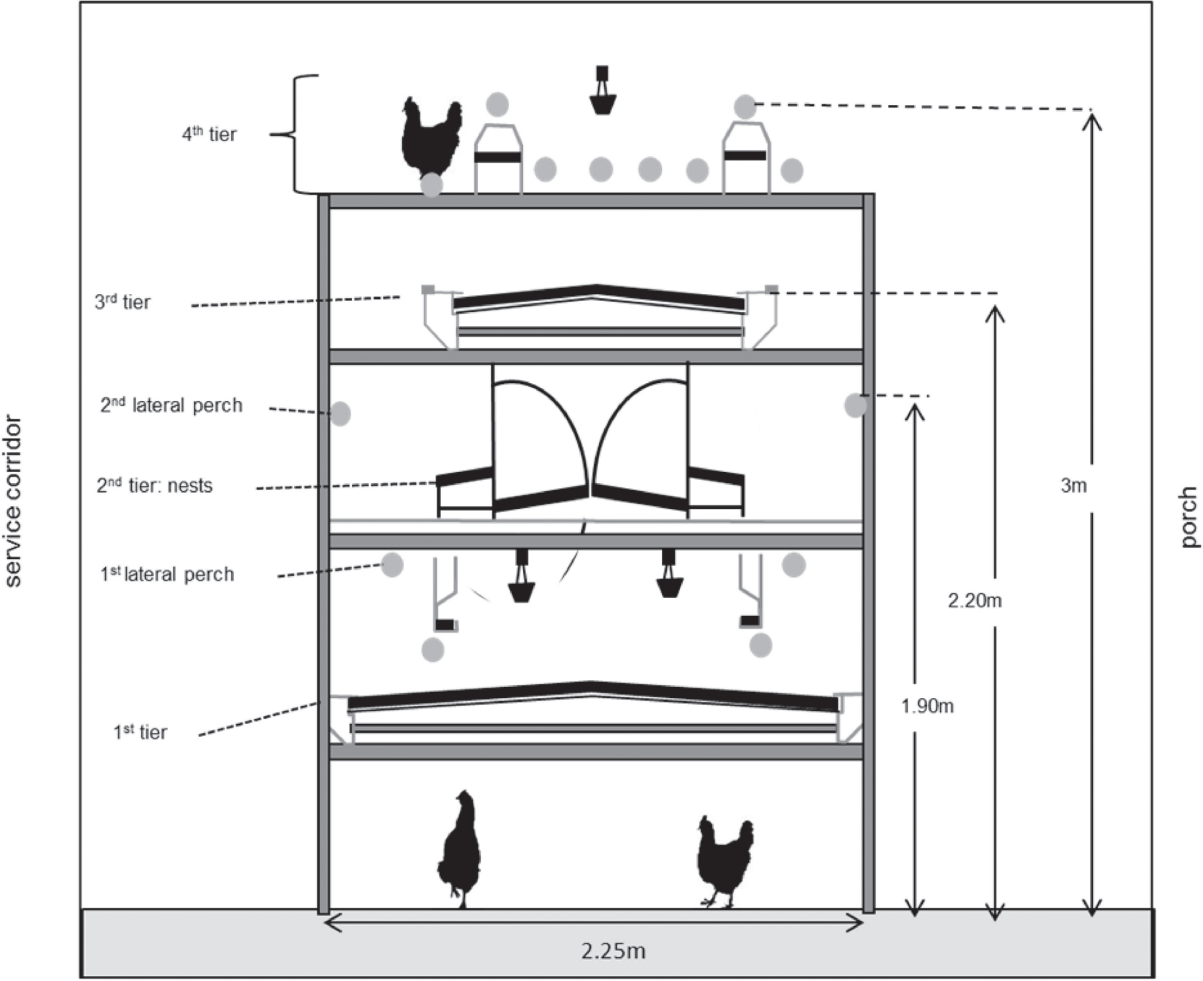
Schematic side view of aviary system Bolegg Terrace.

In total 2295 white (Dekalb White = DW) and 2295 brown (ISA Warren = ISA) chicks were raised separately from hatch until 18 weeks of age in a rearing barn that was divided into eight separate pens equipped with two different aviary systems (four pens with Inauen Natura, Inauen AG, Appenzell, Switzerland and four pens with Landmeco Harmony, Globogal AG, Lenzburg, Switzerland). All pens were equipped with round, metal perches, nipple drinkers, automatic feeding chains and manure belts. Half of the birds had access to a porch (two pens ISA and two pens DW, all Inauen Natura). At 18 weeks of age the hens were moved to a commercial laying hen house where they were distributed across 20 pens (each measuring 450 x 700 x 230 cm). In each pen, 225 individuals were housed and banded with a leg band of a pen-specific colour. Hens were allocated to the pens of the laying hen house in a balanced manner according to hybrid and rearing conditions (i.e. aviary type and porch access) in such a way that birds from different rearing aviaries as well as hybrids were not mixed. The laying hen house was equipped with a 4-tiered aviary (Bolegg Terrace, Krieger AG, Ruswil, Switzerland) that was placed in the middle of each pen, resulting in a stocking density of 7.4 hens/m^2^ accessible floor (including all grid areas and floor). The aviary system was three meters high and consisted of the following furniture across four tiers: a manure belt, nipple drinkers and a feeding chain (1^st^ tier); group nests (2^nd^ tier); another manure belt (3^rd^ tier); and perches, nipple drinkers and a feeding chain (4^th^ tier; Fig. 1). The pen floor was initially covered with wood shavings (approximate depth = 10 cm for both beddings on both sides and underneath the aviary system) and resupplied above the old bedding approximately every two weeks. Each pen had access to a separate outside porch (area = 9.32 m^2^) covered with wood shavings and equipped with perches and nipple drinkers. All perches (16 perches per pen, each measuring 230 cm in length) were round and made of metal with an outer diameter of 3.2 cm. Two perches were provided at both sides of the aviary for changing to other levels (1^st^ lateral perch at 125 and 2^nd^ lateral perch at 190 cm height) and ten perches on the top tier for roosting at night. In 10 pens, all perches were covered with a soft polyurethane material (total outer diameter: 6.6 cm (treatment)) while the perches of the remaining pens were not altered (control). The two experimental factors—hybrid and perch type, each with two levels—were crossed resulting in four treatment combinations across the 20 pens (n = 5 pens per treatment combination). Light was provided by artificial light from 02:00 to 17:00 with a transitional period of 10 min at 02:00 and 20 min at 16:40–17:00. Natural daylight was provided through windows and controlled by curtains from 08:00 to 16:30. The average light intensity in the middle of the pens at 13:00 and 16:50 was 50 and 8 lux, respectively.

### Collected measurements

#### Palpation.

At 18, 21, 23, 30, 38, 44 and 64 weeks of age the keel bones of 20 hens per pen were examined by palpation. Ages were chosen based on Käppeli et al. [22] where palpations were conducted at similar ages to demonstrate the development of keel bone damage with age. Birds were selected while roosting during darkness in a stratified manner (10 hens per pen side and every third hen per tier; 400 birds in total per age) and palpated in the pens, thus the examiner was aware of the perch material and the genetic line. All palpations were performed by one person (A.S.). Birds were not labeled individually and thus not followed longitudinally over the study. The palpation procedure involved running two fingers along the carina sterni and at the side of the keel bone, feeling for malformations that may indicate previous damage [23]. Fractures and deviations were assessed using a scoring scheme from Scholz et al. [11]. In their study, histological examinations were conducted to develop a 4-point scoring scheme ranging from severe (score 1), moderate (score 2), slight (score 3) and no deformation (score 4) indicating the severity of keel bone damage. Palpation in the current study was conducted using these scores but condensed to the following three categories:

- NK (normal, non-damaged keel bone): no signs of fracture or deviation present, no injuries or swellings,
- DK (deviated keel bone): slight deviation of the keel bone from a theoretical two dimensional straight plane including depressions on the ventral surface and/or S-shaped bending in the median sagittal plane
- FK (fractured keel bone): presence of sharp edges, distinct displacements, and bumps assumed to indicate the presence of callus material and suggesting fractures [24].

If a keel bone had both a fracture and a deviation, the bird was assigned to the FK category, assuming fractures were more likely to be associated with pain than deviation and thus more of a welfare concern. This assumption is based on the reasoning that keel bone deviations likely result from a protracted process (i.e. bone remodeling in response to continued and relatively consistent pressure [25]) rather than a short-duration trauma as with fractures [11]. The FK category represents a combination of the scores 1 and 2 used by Scholz et al. [11]. For the last palpation (64 weeks of age) 200 hens (10 per pen) were selected and palpated while roosting during darkness as described above. The other 200 birds (10 per pen) were caught during daylight after dimming the light, evenly chosen from each tier and both sides of the aviary, transferred into transport boxes, and palpated in a different room of the barn (blinded assessment). These 200 hens were marked with a numbered wing tag, transported to the abattoir, slaughtered, the keel bones were removed with a secateurs, cleaned of excess flesh and stored with the wing tag for identification in a freezer at—21°C. To validate the palpation method and measure accuracy of the live palpation, the collected keel bones were defrosted later and visually assessed for fractures and deviations using the described scoring system for comparison with palpation data collected on the same birds.

#### Feather score and body mass.

At 21 and 44 weeks of age, feather condition of 10 hens per pen, randomly selected from different tiers of the aviary, were scored [26] using a scale of 4 (full feather cover) to 1 (severe damage). Five different body parts per hen were assessed including neck, back, wings, tail and ventral side. Body mass of each palpated hen was recorded at 30 and 64 weeks of age to the nearest 20 g with a spring balance (Pesola AG, Baar, Switzerland).

#### Production measures.

Beginning at 18 and continuing until 64 weeks of age, daily egg production, feed consumption, floor eggs and mortality were recorded for each pen. Egg mass was recorded twice per month from 18 until 44 weeks of age and once per month from 45 until 64 weeks of age for each pen. Laying performance was calculated as the total eggs laid per pen per day divided by the number of live birds. Daily feed consumption per egg was calculated as total feed consumption per pen divided by the number of eggs laid per pen and day. Floor eggs as a percentage of total number of eggs laid and mortality as a percentage of total number of birds per pen were calculated daily. Egg mass was calculated as the total egg mass divided by the number of total eggs laid per pen.

### Statistical Analysis

#### Palpation.

To assess overall incidence of keel bone damage and the relationship to perch type and genetic line, we used logistic regression to compare the absolute number of bones assigned to categories DK and FK (damaged keel bones) with the absolute number of bones assigned to category NK (non- damaged keel bones) by analyzing the data as a binary response variable. To assess incidence of fracture, we compared the absolute number of bones assigned to category FK (fractured keel bone) with the number of bones assigned to the categories DK and NK (non- fractured keel bone) in a second analysis. For both analyses, data were analyzed using a generalized linear mixed model (RStudio version 3.0.1, package ‘lme4’, function ‘glmer’, family = binomial). Prediction variables were hybrid (ISA/ DW), perch type (hard/ soft) and age (18, 21, 23, 30, 38, 44, 64 weeks of age as a continuous variable). Pen was treated as a random factor on which repeated measurements (i.e. week of age) were taken. Porch access during rearing (yes/ no) was initially included in both models and checked whether it related to each of the category of keel bone assignments. Since rearing style was not expected to influence responses and no effect was found in either analysis, porch access during rearing was excluded in further analyses. A third analysis was conducted to assess the relationship between severity of keel bone damage and predictors, using the assigned scores of keel bone status (NK vs. DK vs. FK) as the response variable in a multinomial logistic regression (package: ‘mlogit’, function: ‘mlogit’) with perch type and hybrid as predictors. In this model, the likelihood of FK or DK being assigned was compared with NK for each predictor. For each of the three analyses, resulting coefficients were used to calculate odds ratios for each of the associated predictors of the final model. Palpations were conducted in a manner that the assessor was aware of treatment except at the last palpation date at 64 weeks of age, where 200 birds were palpated blinded and 200 birds were palpated while being aware of the treatment. Since being aware of the treatment may be a confounding factor we compared the two palpation methods for both response variables (damaged vs. non-damaged keel bone; fractured vs. non-fractured keel bone) using blindness as a fixed factor (yes/no) in a separate analysis. The analysis was conducted for the last palpation date only (64 weeks of age) and included the fixed factors perch type (hard/soft), blindness (yes/no), and hybrid (ISA/DW). Data values were considered significant at p ≤ 0.05. Non- significant interactions were pooled (p > 0.2).

#### Feather score and body mass.

Scores of the five different body parts were pooled per hen and the mean scores per bird were again pooled per pen and age (assessed at 21 & 44 weeks of age). Feather score was not distributed normally, so was transformed into a binary response variable by grouping score 1, 2 and 3 and compared against score 4.

The relationship between body mass and predictors (hybrid, perch type and age as a categorical variable) was analyzed at 30 and 64 weeks of age using a general linear model (package ‘lme4’, function ‘glm’, family = gaussian) after checking for a normal distribution using histogram plots for each factor and Q-Q plots of the residuals. Data values were considered significant at p ≤ 0.05. Non- significant interactions were pooled (p > 0.2).

#### Production data.

Production data were summarized for the entire laying cycle beginning 18 until 64 weeks of age separated for each pen. Data were analyzed using a general linear model (package ‘lme4’, function ‘glm’, family = gaussian) after testing for normal distribution using histogram plots for each factor and variable. Fit of the model was examined using Q-Q plots of the residuals. Predictors were hybrid (ISA/ DW) and perch type (hard/ soft). All response variables (laying performance (%), egg mass (g), feed consumption/egg (g), floor eggs (%) and mortality/ laying period (%)) were normally distributed. Data values were considered significant at p ≤ 0.05. Non- significant interactions were pooled (p > 0.2).

#### Intra- and inter observer- reliability.

We assessed the intra- and inter observer-reliability of the scoring method using Cohen’s kappa coefficient (0 ≤ κ ≤ 1) as an index of inter-rater agreement (package ‘psych’, function ‘cohen.kappa’). To evaluate intra-observer reliability and repeatability of the scoring method, a sample of birds not part of the current experiment was palpated twice (n = 123) with a two hour interval in between. Analysis identified a κ- score of 0.7 (95% CI [0.59, 0.81]), which is valued as substantial [27]. To evaluate inter-observer reliability, a second group of birds from another experiment (n = 44) were palpated by A. S. and S.G. after the end of the present experiment yielding a κ- value of 0.54 (95% CI [0.35, 0.74]), which is valued as moderate [27]. The conformance between live palpation and visual assessment of the keel bones that were collected and cleaned after slaughtering was assessed as moderate (κ- value: 0.53, 95% CI[0.41, 0.64], N = 139) [27]. The accuracy in terms of percentage of correct assessments between live palpation and visual keel bone assessment was 68%.

## Results

### Palpation

**Fig 2:**
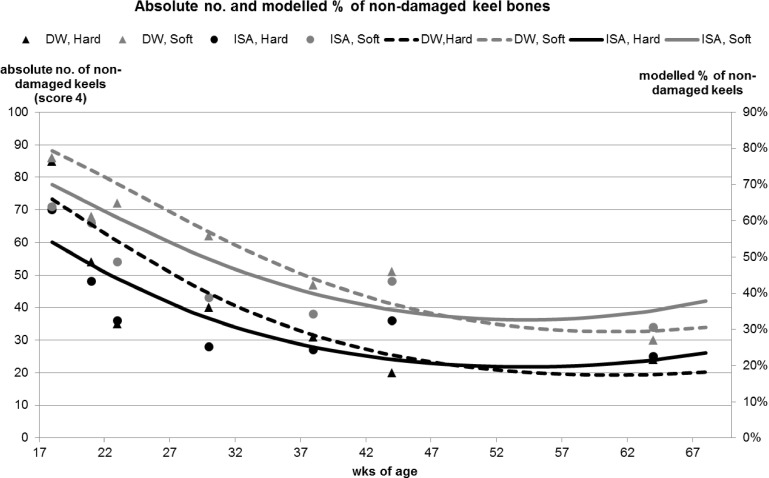
Non-damaged keel bones. Absolute numbers and modelled % of birds with a non-damaged keel bone over the course of the experiment for each of the four perch type by genetic line combinations.

**Fig 3:**
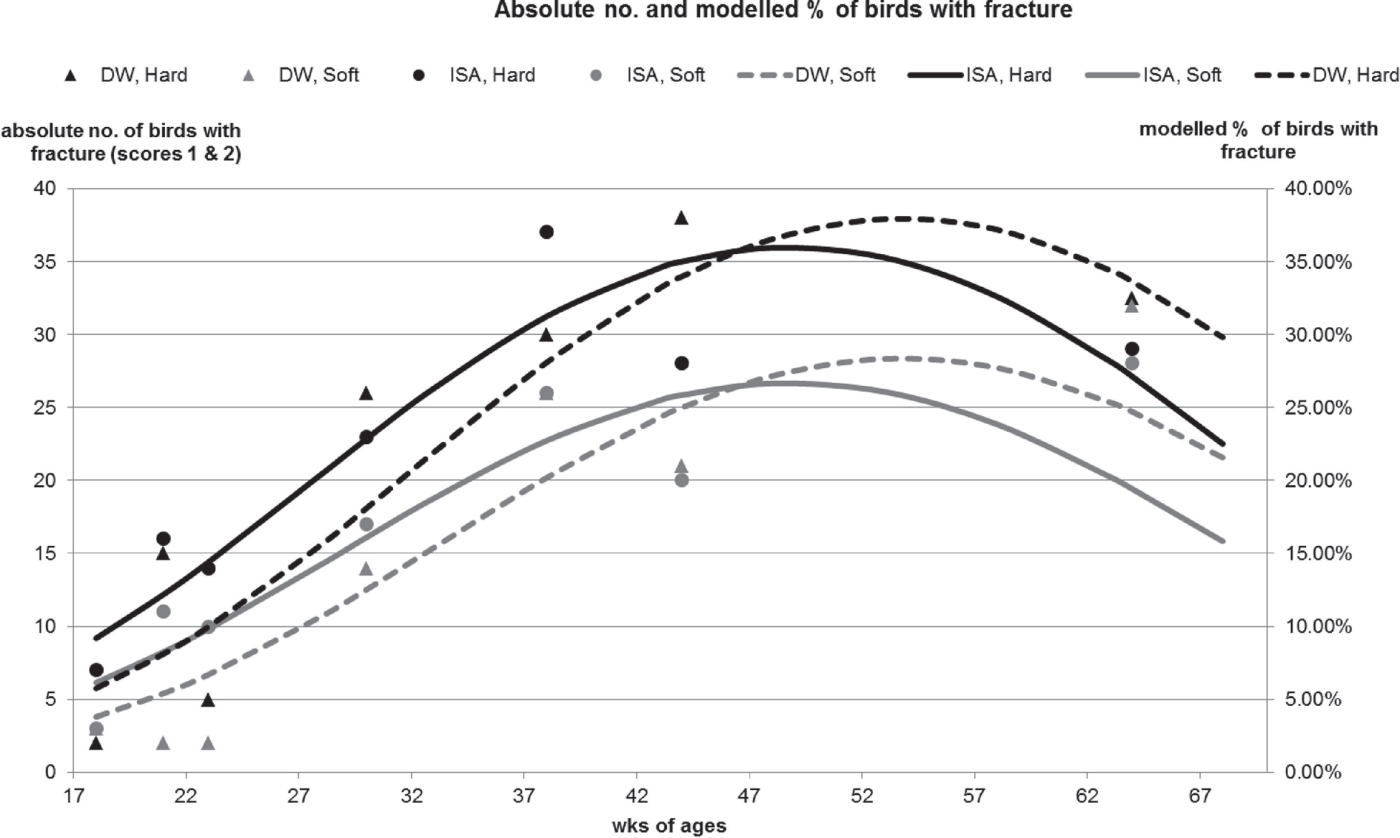
Fractured keel bones. Absolute numbers and modelled % of birds with a fractured keel bone over the course of the experiment for each of the four perch type by genetic line combinations.

The first analysis of palpation data focusing on overall keel bone damage (damaged (FK/DK) vs. non-damaged keel bone (NK)) revealed that pens with soft perches had a greater percentage of birds with non-damaged keel bones (Z = 7.06, p< 0.0001; averaged percentage of non-damaged keel bones for entire experiment: soft 55.1% vs. hard 39.8%). An age by hybrid interaction was identified revealing that at the initial observation (18 weeks of age) more birds of the DW hybrid had a non-damaged keel bone compared with birds of the ISA hybrid. However at later observations (44 and 64 weeks of age) the percentage of non-damaged keel bones decreased and fewer birds of the DW hybrid had a non-damaged keel bone compared with birds of the ISA hybrid (Z = 2.97, p = 0.003). A modeled representation of the data is shown in Table 1 and Fig. 2. The dataset is available in S1 Data. The second analysis, focusing on fractures as the type of keel bone damage (fractured (FK) vs. non-fractured keel bone (DK/NK)) revealed that pens with soft perches had a lower percentage of birds with fractured keel bones (Z = -3.25, p = 0.0012; averaged percentage of fractured keel bones for entire experiment: soft 15.4% vs. hard 21.5%). An age by hybrid interaction was identified revealing that at the first observation (18 weeks of age) more birds of the ISA hybrid had a fractured keel bone compared with the DW hybrid. This trend was reversed at later observations (44 and 64 weeks of age) where more birds of the DW hybrid had a fractured keel bone compared with the ISA hybrid (Z = -2.33, p = 0.02). A modeled representation of the data is shown in Table 2 and Fig. 3. The third analysis, focusing on different types of keel bone damage (NK vs. DK vs. FK) showed that pens with soft perches had a reduced percentage of birds with deviated and fractured keel bones compared with non- damaged keel bones (DK: b = -0.6, p < 0.0001; FK: b = -0.66, p < 0.0001). The odds ratio revealed that assignment to DK (deviation) and FK (fracture/ deviation) was 1.8 times and 1.9 times more likely to occur in pens with hard compared with soft perches, respectively. Also, the ISA hybrid had an increased likelihood of being assigned to DK and FK compared to NK (DK: b = 0.22, p = 0.04; FK: b = 0.23, p = 0.007). The calculated odds ratio showed that assignment to DK and FK was both 1.3 times more likely to occur in the ISA hybrid than in the DW hybrid. Blindness did not affect the results of the last palpation at 64 weeks of age (NK vs. DK/FK: Z = -0.89, p = 0.37; FK vs. DK/NK: Z = 1.63, p = 0.103). Interactions between perch type by blindness and hybrid by blindness were both non- significant. Also, independent of being aware or blind, no difference between the four different treatment groups was found at 64 weeks of age for damaged vs. non-damaged keel bones (perch type: Z = 1.78, p = 0.075; hybrid: Z = 0.67, p = 0.50) and fractured vs. non-fractured keel bones (perch type: Z = -0.11, p = 0.91; hybrid: Z = -0.76, p = 0.45).

**Table 1:** Model output for the likelihood of non-damaged keel bones occurring.

	Likelihood of non-damaged keel bones (binary)		
Term	Estimate	SE	Ref ^†^
β^0^	2.91	0.31	
Hybrid	-0.79	0.21	ISA
Age	-0.15	0.02	
Perch type	0.68	0.1	soft
Hybrid: Perch type	0.02	0.01	

^†^For this analysis, the category in the `Ref`column serves as the reference from which the other category is compared against (Hybird ISA vs. DW; Perch type soft vs. hard).

**Table 2:** Model output for the likelihood of keel bone fractures occurring.

	Likelihood of keel bone fracture (binary)		
Term	Estimate	SE	Ref ^†^
β^0^	-5.7	0.45	
Hybrid	0.82	0.29	ISA
Age	0.19	0.02	
Perch type	-0.43	0.12	soft
Hybrid: Perch type	-0.02	0.01	

^†^ For this analysis, the category in the `Ref`column serves as the reference from which the other category is compared against (Hybird ISA vs. DW; Perch type soft vs. hard).

### Feather score and body mass

Body mass differed between the hybrids (t = 9.013, p < 0.001) but was not affected by perch type. An interaction for body mass was found between hybrid and age (t = -2.174, p = 0.03). The ISA hybrid was heavier at 30 than at 64 weeks of age and heavier than the DW hybrid at both ages (raw data: 30 weeks of age: ISA hybrid: 2098 g ± 158.5, DW hybrid: 1847 g ± 124.7; 64 weeks of age: ISA hybrid: 2036 g ± 196.9, DW hybrid: 1835 g ± 159.2). The ISA hybrid had a poorer feather condition at 44 but not at 21 weeks of age compared with the DW hybrid (Z = 2.42, p = 0.016). The dataset is available in S2 Data.

### Production data

The DW hybrid laid more eggs and consumed less food per egg compared with the ISA hybrid (Table 3). The ISA hybrid laid heavier eggs in pens with soft perches compared with the DW hybrid, which laid heavier eggs in pens with hard perches (Table 3). The percentage of floor eggs was higher in pens containing ISA hybrids and soft perches than in pens containing DW hybrids and hard perches (Table 3). Mortality was higher in the ISA hybrid compared with the DW hybrid (Table 3). The dataset is available in S3 Data.

**Table 3:** Production data for genetic line (DW/ ISA) and perch type (hard/ soft), N = 20.

	Hybrid				Perch type				Hybrid x Perch type
	ISA	DW	SEM	P-value	Hard	Soft	SEM	P-value	P- value
**Laying performance (%)**	83.60	93.73	0.46	< 0.001	89.15	88.18	1.74	0.182	0.1
**Egg mass (g)**	66.7	64.2	0.1	< 0.001	65.4	65.5	0.42	0.169	0.032
**Feed consumption (g)**	145.0	126.7	0.75	< 0.001	134.7	136.9	3.13	0.868	0.068
**Floor eggs (%)**	3.47	1.82	0.27	0.113	2.77	2.52	0.38	0.042	0.029
**Mortality/ laying period (%)**	1.37	0.25	0.11	< 0.001	0.88	0.74	0.23	0.489	0.489

## Discussion

To our knowledge this is the first published evidence of the effectiveness of a soft perch material to reduce keel bone damage in a commercial laying hen operation. As expected, our results show that soft perch material reduced the number of damaged keel bones—both deviated and fractured keel bones. As an explanation to this change, short-duration trauma such as high energy collisions are assumed to be one of the main causes for the high incidence of keel bone fractures, particularly in aviary systems. Pickel et al. [12] hypothesized that soft perches absorb energy released during high energy impacts (e.g. collisions with perches) resulting in a reduced number of fractured keel bones. Our results support this hypothesis and provide a possible mechanism for the reduction of keel bone fractures in aviary systems. Soft perches may convert kinetic energy that occurs during collisions into deformation energy as the cushion is compressible and thus reduces the energy the keel bone is required to absorb. The system is comparable to an automobile’s shock absorption system. Without shock absorption (e.g. hard perch material), little kinetic energy is absorbed and energy is instead transferred undamped to the bone and thus might cause fractures [28]. In humans, external soft protectors are used to prevent hip fractures in elderly people suffering from osteoporosis and have been shown to reduce risk of hip fractures by 60% [29] indicating the benefit of soft material in comparable situations. The surface of the soft perch might also provide a tactile benefit to the birds [12] allowing for improved traction and providing a better grip. Therefore in addition to a reduced likelihood of injury when collisions occur, the overall number of falls and associated collisions could be reduced as well.

Soft perches appeared to benefit the keel bones also with less incidence of deviation occurring. This could be explained by an enlarged contact area between the keel bone and the perch providing increased spread of pressure on the keel bone while perching. Evidence in support of this mechanism has been reported by Pickel et al. [12], who tested similar perch materials and found that peak force on the keel bone was lower and contact area between keel bone and perch was larger in soft, air-cushioned perches compared with standard, hard perches. Keel bone deviations are assumed to result from extended perching duration since hens mainly seem to place a major portion of their mass on their keel bone while perching [12]. In addition, local pressure load in combination with the thin anatomical structure and exposed location of the keel bone may increase the keel bones’ susceptibility for deviations. Assuming that a minimum peak force and a maximum contact area are beneficial for keel bones, this mechanism may explain the reduced number of deviations seen in the soft perch pens. A potential confound between perch types is that the diameter of the soft perch was double the size of the hard perch. An increased perch diameter may provide better footing and reduce balance movements [30], resulting in less falls and collisions. Larger perch width is also preferred compared to thinner ones and may have affected their use as well [31]. Thus, in the current study the different perch diameter as well as the material might both have influenced the effect of soft perches on keel bone fractures and deviations. Future research using a combined approach of perch diameter and material will be needed to clarify this effect.

Palpation at different ages revealed that the percentage of sampled birds with a fracture increased with age, a fact that is consistent with other studies [22, 32, 33]. In contrast to these studies, we found that the proportion of sampled bird with fractures did not increase beyond 52 weeks of age. The lack of a further increase was unexpected, though has been found by others [10] and could be related to several mechanisms. One potential mechanism is altered behaviour such as less activity and/or better navigation of the housing environment with increasing age (*unpublished data*). A second mechanism is changed bone properties such as flexibility and/or strength, a finding supported by increased strength in the keel bone over time (*unpublished data*), though this pattern is inconsistent with bone strength reduction [34] or no change [35]. Changes in bone properties is supported by an ex vivo model in euthanized birds [36], which suggested that factors independent of behaviour, e.g. bone health, resulted in a decreased fracture susceptibility at approximately 45 weeks of age. The ambiguity of these results suggest that further research is needed to better understand changes in bone properties and bone remodeling with increasing age. In the current experiment the pattern of fractures occurring over the course of the laying period was similar for all treatment groups with the exception of pens with soft perches where fewer keel bone fractures were detected. However, at the end of the experiment (64 weeks of age) no difference between the treatment groups regarding number of fractured keel bones was detected (both perch types 30% fractures), indicating that there were limits to the benefits of soft perches. Therefor our results suggest that other factors including housing design and genetic selection need to be considered as well to reduce keel bone damage in laying hens, particularly towards end of lay.

Differences between hybrids regarding incidence of non-damaged and fractured keel bones were dependent on age. Around onset of egg production (18 to 21 weeks of age), the ISA hybrid had more fractures and fewer non-damaged keel bones compared with the DW hybrid. Contrastingly, at the end of the experiment more DW hybrids were assigned to the category FK and fewer birds to category NK compared with the ISA hybrid. Differences between layer lines regarding keel bone damage have been described in several studies [37, 38, 12, 39], though were not found by Käppeli et al. [22] or Gebhardt-Henrich & Fröhlich, [40]. The brown hybrid lines were more susceptible to fractures compared with white layer lines [38, 12], though findings are inconsistent [39]. The notion that body mass is related with incidence of fracture is supported by a higher incidence of fractured keel bones in the ISA hybrid, which was also heavier than the DW hybrid. In particular, a greater body mass could cause greater collision energies when the hen is colliding with a perch. Greater body mass might also increase pressure on the keel bone while perching and cause more deviations. A higher incidence of fractured keel bones in the DW hybrid at the end of the experiment might be related with an increased activity of the DW hybrids throughout the experiment, which panicked more easily than the ISA hybrid (pers. observation), which is likely to result in more flight accidents and collisions over the course of the experiment. However neither perch material nor genetic line influenced number of falls around dusk and darkness at 18 and 43 weeks of age (data not shown).

Practicability of the soft perch material was reduced as problems concerning hygiene, parasites and fixation to the metal perches occurred at around two months after beginning of the experiment. Fixation of the soft material to the metal perches needed to be maintained regularly with cable ties. The material also harboured red mites, in particular during warmer months, which is likely to increase stress level and possibly even increase mortality in laying hens [41]. Therefore the usability of this particular soft material as it was tested in this study must be considered in light of bird hygienic and related health issues. Nevertheless, as our results suggest, soft material seems a valid means to reduce keel bone damage if adjusting it for large scale hen housing systems.

In this study the categories used to assess keel bone damage were adopted from Scholz et al. [11], who developed a scoring method to assess severity of keel bone damage by using four different scores. Histological examinations of the different scores revealed that the score indicating slight keel bone deviation (score 3) included 50% bones with callus material, indicating a fracture, and 50% bones without callus material but a deviation. The category deviated keel bone (DK) in our study would represent score 3 from the system used by Scholz et al. [11] and thus we cannot exclude the possibility that some of the bones assigned to category DK should actually be assigned to the category FK, which is also indicated by the lower agreement between live palpation and visual assessment of dissected keel bones as more fractures were found in the visual assessment of the bones compared with live palpation. Conformance between live palpation and visual keel bone assessment was 53% only which was due to live palpation underestimating visual keel bone assessment since especially small keel bone fractures or fractures at difficult sites to palpate such as the tip were missed during live palpation. A combination of live palpation and visual keel bone assessment throughout the experiment including killing a small sample of birds in each palpation phase would have improved reliability of assessing keel bone damage in this study.

## Conclusion

We report that soft perch material has the potential to reduce incidence of keel bone fracture, probably by lowering the kinetic energy on the keel bone during collisions. Secondly, the number of deviations was reduced suggesting that the soft perch material provides an increased spread of pressure on the keel bone during perching. Although throughout the entire laying period cushioned perches reduced keel bone fractures and deviations, at the end of laying period the incidence of fractured keel bones converged. Thus, additional measures including genetic selection for more resilient bones, modified feeding and improved housing design should be considered to further reduce keel bone damage in laying hens.

## Supporting Information

S1 File ARRIVE Guidelines Checklist. (DOC)

S1 Data Dataset keel bone damage. (XLSX)

S2 Data Dataset body mass. (XLSX)

S3 Data Dataset production. (XLSX)
